# Family doctors' problems and motivating factors in management of depression

**DOI:** 10.1186/1471-2296-7-64

**Published:** 2006-10-30

**Authors:** Pille Ööpik, Anu Aluoja, Ruth Kalda, Heidi-Ingrid Maaroos

**Affiliations:** 1 Department of Family Medicine, University of Tartu, Ülikooli St. 18, 50090 Tartu, Estonia; 2 Department of Psychiatry, University of Tartu, Tartu, Estonia

## Abstract

**Background:**

Depression is a frequent psychiatric disorder, and depressive patient may be more problematic for the family doctors (FD) than a patient suffering from a somatic disease. Treatment of patients with depressive disorders is a relatively new task for Estonian FDs. The aim of our study was to find out the family doctors' attitudes to depression related problems, their readiness, motivating factors and problems in the treatment of depressive patients as well as the existence of relevant knowledge.

**Methods:**

In 2002, altogether 500 FDs in Estonia were invited to take part in a tailor-made questionnaire survey, of which 205 agreed to participate.

**Results:**

Of the respondents 185(90%) considered management of depressive patients and their treatment to be the task of FDs. One hundred and eighty FDs (88%) were themselves ready to deal with depressed patients, and 200(98%) of them actually treated such patients. Commitment to the interests of the patients, better cooperation with successfully treated patients, the patients' higher confidence in FDs and disappearance of somatic complaints during the treatment of depression were the motivating factors for FDs. FDs listed several important problems interfering with their work with depressive patients: limited time for one patient, patients' attitudes towards the diagnosis of depression, doctors' difficulties to change the underlying causes of depression, discontinuation of the treatment due to high expenses and length. Although 115(56%) respondents maintained that they had sufficient knowledge for diagnostics and treatment of depression, 181(88%) were of the opinion that they needed additional training.

**Conclusion:**

FDs are ready to manage patients who might suffer from depression and are motivated by good doctor-patient relationship. However, majority of them feel that they need additional training.

## Background

Depression is one of the most frequent psychiatric disorders playing a significant role in the overall morbidity of population. In Europe, up to 10% of the population and 25% of the primary health care patients suffer from depression [1,2]. An Estonian survey revealed that 11.1% of the population had symptoms of depression [3]. In 1990, depression was the fourth most frequent cause of disability [4]. It is prognosticated that by 2020 depression will be the second most common cause of disability after cardiovascular diseases [5]. At the same time, a depressive patient may be more problematic for the FD than a patient suffering from a somatic disease [6,7]. Treatment of depressive patients takes more time [8,9]. Diagnosis of depression often presents difficulties, as it is time-consuming and requires more clinical investigations [10]. The ability of FDs to diagnose and treat depression is directly related to their knowledge and further training [11]. Only a few studies have focused on the FDs' opinion about depression related problems in their work [11-13]. This disease is a heavy burden for primary health care and therefore prevention strategies should be worked out. In 2003 the international project PREDICT was launched to obtain knowledge of the risk factors in order to make use of it in depression prevention [14]. Carrying out such a comprehensive survey and using its results in primary care demands active participation of FDs and readiness to deal with depressive patients. The present survey has been designed to learn the promoting and restricting factors in order to fulfil this task. Besides, it served as a background study for the PREDICT project in order to clarify the readiness and willingness of FDs to take part in it.

The aim of our study was to find out the FDs' readiness, motivation and problems in treating depression as well as the need for further training.

## Methods

A questionnaire-based survey was conducted during two months in 2002. The tailor-made questionnaire included both closed and open questions. The questionnaire consisted of 10 questions (Appendix 1). The closed questions required yes/no answers. The open questions required a description of an opinion such as: What is your motivation in dealing with depressive patients? What problems do you face in your everyday work with depressive patients? The questions were divided into five groups: 1) FDs' background: location of the practice (urban/rural), solo or group practice, age, gender, length of service; 2) FDs' readiness to deal with depressive patients and their competence; 3) FDs' motivation to deal with depressive patients; 4) FDs' opinions about depression related problems; 5) FDs' self-evaluation of the knowledge of depression and the need for further training.

The questionnaires were sent by post to 500 (89% of all FDs) certified practising FDs in Estonia in October 2005. To increase the response rate, repeat questionnaires were delivered two months later to all FDs. Two hundred and five questionnaires were returned. The results were analysed with the use of frequency distribution tables. The differences between the groups were tested using the Chi-square test. The open questions were analysed using thematic analysis [15,16]. All answers to open-ended questions were recorded. The subsequent statements were first analysed by one of the authors (ÖP) for identifying any statements related to FDs' motivation in order to deal with depressive patients and problems arising during work with depressive patients. All statements expressing motivation for, or indicating problems in working with depressive patients were coded and categorized according to their content and the categories were labelled in order to verify that the described findings reflected the database adequately. The second author (AA) analyzed the texts independently in a similar way. A few ambiguities in the analyses were discussed to reach consensus.

## Results

Of the respondents 84(41%) worked in rural and 121(59%) worked in urban areas. The background of the FDs, who received the questionnaire, has been given in table 1.

**Table 1:** Background characteristics of the respondents

Location	Solo practice n (%)	Group practice n (%)	Average age, years (± SD)	Average length of service as a physician, years (± SD)	Average length of service as a FD, years (± SD)
Rural (n = 84)	63(75)	21(25)	45.9(± 8.4)	19.1(± 7.7)	5.1(± 1.3)
Urban (n = 121)	35(29)	86(71)	45.7(± 8.5)	19.5(± 9.2)	4.5(± 2)
Total (n = 205)	98(48)	107(52)	45.8(± 8.5)	19.4(± 8.6)	4.8(± 1.5)

There were no differences between the characteristics of the sex and location of the practising FDs and the respondents in this study (table 2).

**Table 2:** Comparison of the characteristics of the respondents

	Total n	Location		Sex	
		
		Rural n(%)	Urban n(%)	Female n(%)	Male n(%)
FDs who were asked to participate in the survey	500	215(43)	285(57)	469(94)	31(6)
FDs who completed the questionnaire	205	84(41)	121(59)	188(92)	17(8)

No differences between the characteristics of the FDs who were asked to participate and those who completed the questionnaire (p > 0.05)

Most of the FDs were willing to deal with and manage depressive patients and considered it being within their competence (table 3). Those, who did not deal with depressive patients, explained it mainly due to lack of relevant knowledge, high prices of drugs and their indeterminate effect. They considered the problems of such patients to be solely the responsibility of psychiatric service.

There were no significant differences between the responses given by the FDs working in urban and rural practices (p > 0.05), or in solo and group practices (p > 0.05) (table 3).

**Table 3:** FDs' opinions about depression management depending on the location and character of their practice

	Total	Location		Type of practice	
		
		Rural	Urban	Solo	Group
Is it your daily work? Yes n (%)	185(90)	72(89)	107(91)	86(91)	93(89)
Are you ready to deal with it? Yes n (%)	180(88)	71(89)	104(88)	83(88)	92(88)
Do you deal with depression? Yes n (%)	200(98)	80(99)	114(96)	93(98)	101(97)

There was no significant difference in the studied characteristics between the rural and urban, solo and group practices (p > 0.05)

The motivation factors for the FDs were grouped into five topics according to the content.

1. High prevalence of depression in primary health care

"Depression is widespread." "There is a great need for depression treatment." "Depression often accompanies the main disease."

2. Family doctors' feeling of commitment

"I hope I can help patients." "We cannot be dispatchers sending people to various places." "Patients refuse to see a psychiatrist; FDs have to manage on their own."

3. Positive results of treatment

"After effective treatment the patient is reborn." "Earlier positive experience in depression management." "If patients receive help, further co-operation will be good." "If treatment is effective, the patient will not demand clinical investigations any more." "Several somatic complaints cease during the treatment of depression." "Patient's recovery gives much satisfaction."

4. FDs' advantages

"Patients' trust is important." "We know our patients better than psychiatrists do." "FDs are better informed of the concomitant diseases." "It is much easier for the patient to consult the FD."

5. Convenience from the patient's point of view

"A bedridden patient at home cannot go anywhere else." "Patients do not want to see the psychiatrist. Psychiatrists' offices are located far from the patients' homes." "Specialists' waiting lists are long."

The problems that the family physicians described during the time they dealt with depressive patients' were grouped as follows:

1. High rate of depression in primary health care

"Patients' depressive disorders are a daily problem." "Almost every day we can see a patient with a depressive background." "Depression has become more widespread over the years."

2. High cost of management of depression

"The depressive patient requires more time for consultation to delve in psychological problems." "Depression is often accompanied by multiple somatic complaints and patients place high expectation on the investigations performed with the use of apparatuses."

3. Patients' difficulties with accepting the diagnosis and with the subsequent treatment

"It is difficult to explain to the patient that depression is the cause of all his/her complaints." "Patients feel that somatic diseases are "respectable" diseases and are afraid to accept the diagnosis of depression." "Patients refuse to see the psychiatrist because they think of them as shrinks who treat insane persons." "Patients do not recognise the cause of depression; they ignore it and will not do anything to change the situation." "Many patients stop taking their medication or do not start altogether because drugs are expensive." „Psychological counselling is expensive and psychotherapy is unavailable for many persons due to the location of their home."

4. Physicians' inadequate resources/skills to help patients

"Sometimes FDs do not recognise depression." "It is difficult or impossible for the physician to eliminate the causes of depression." „In addition to drugs, patients need psychotherapy, behavioural therapy, family therapy, etc.; however, we do not have such skills." "It is difficult to refer patients to psychiatrists due to their long waiting lists." "Seeing the psychiatrist often involves additional costs for patients as psychiatric aid may not be available in the neighbourhood." "There is no co-operation between the FD and the psychiatrist or the psychologist."

Concerning sufficiency of the knowledge to deal with depression, or the need for further training, 115(56%) respondents claimed to have sufficient knowledge to diagnose and treat depression, and 90(44%) respondents considered their knowledge inadequate. The opinion of 181(88%) physicians was that they definitely needed further training and 24(12%) of them maintained that they did not need any training.

FDs' assessment of their knowledge of depression and the need for further training is given in table 4.

**Table 4:** FDs' assessment of their knowledge of depression and the need for further training

	Need for further training		Location	
	
	Yes n (%)	No n (%)	Rural n (%)	Urban n (%)
Knowledge is sufficient	95(52)*	21(87)*	39(46)**	76(65)**
Knowledge is insufficient	81(48)	3(13)	45(54)	42(35)

*A significant difference in the opinions about the knowledge between the FDs who felt the need for further training and those who did not p < 0.01

**A significant difference in the opinions about knowledge between the rural and the urban FDs p < 0.01

There was a significant difference (p < 0.01) in the opinions about the knowledge between the physicians, who wanted to receive further training and the ones who did not. The opinions about the knowledge differed significantly between the rural and the urban physicians. The FDs working in towns considered their knowledge to be better compared with those working in the country (p < 0.01).

## Discussion

In the present study we evaluated the readiness and motivation of FDs to manage patients with depressive symptoms within the recently established family medicine system. Depression is a common problem in many countries [1-3]. This situation is new for Estonian FDs. Fifteen years ago the psychiatrist was the person who treated depressive patients. In 1991 training of FDs was started in Estonia. It changed medical service in primary care. The patient can visit the FD free of charge. Every patient is free to attend his/her FD. However, visit fees were introduced in secondary care, including psychiatry [17,18]. FDs can treat patients with psychiatric disorders themselves or refer them to psychiatrists. At the same time a patient can also go to a psychiatrist without a referral from FD. As the FD is usually the first person to see patients with depressive symptoms, it is important to know how well he/she is prepared for this task. The questionnaire used in this study was tailor-made and designed specifically for FDs to display the characteristic features of motivation to deal with the patients' psychological problems. The limitation of the study was the fact that only 41% of the practising FDs agreed to take part. However, as sex and employment characteristics of the respondents corresponded to those of the FDs in general, our results should reflect the real situation regarding the studied problem.

Most FDs considered depression management their task. They were ready to treat and they actually treated such patients in their daily work. The attitude of the FDs did not depend on the location or association of their practice. These results are similar to those obtained in countries with different social and cultural backgrounds [12,19].

Among the motivational factors, two groups of factors are evidently based on the FDs' sense of duty. As depression is highly prevalent in primary care, FDs acknowledge a great need for its treatment. The feeling of commitment is related to the understanding that FDs have primary responsibility for their patients' treatment. Third, FDs feel that successful treatment of depression improves the patient's health as well as doctor-patient relationship. Perception of their advantages such as better knowledge of patients and easier accessibility is another source of motivation for FD's. Advantages are based on patient's interests. In agreement with other studies, FDs feel that patients have more trust in them than in an unknown specialist [8,20]. These results suggest that in treating depression FDs are mainly motivated by patient- and relationship-oriented factors. The results of our study about motivating factors give hope that FDs will pay more attention to prevention of depression in the future. As a result of grouping the problems issues related to the FDs resources and to the patients' attitudes emerged. The responses show again that patients with psychological problems are common. Frequency of depression appeared as an important motivating factor. But it was also mentioned as problematic, because depressive patients increase the workload of FDs. Several studies have shown that 10 to 25 per cent of patients, who visit a primary health care specialist, suffer from depression from time to time [2,6]. Short consultation time per patient is one of the most disturbing factors for FDs in dealing with depressive patients. An average visit to FD lasted 9.0 min according to an Estonian study of consultations. The same study reported that the longest consultation lasted 36.3 min in the case of a psychological problem [9]. So the usual 9-minute consultation is too short a time for dealing with problems of mental health. After the first visit FD can book a longer time for the consultation for patients with psychological problems. A New York study and a New Mexico study reported that visits of depressive patients to their family doctors were longer compared with visits of other patients [8,20]. A Scottish opinion survey showed the importance of consultation time in the outcome of depression treatment [12]. Our results show that the large number of somatic complaints creates diagnostic difficulties; treatment of depression requires time and numerous clinical investigations. Other resources that our FDs considered insufficient were specialized knowledge and cooperation with other specialists. FDs in our study were worried about not being able to eliminate causes of depression and mastering different treatment modalities. Similar characteristics were demonstrated also by other research [10,13,21]. Some problems arise from patients' attitudes and behaviour according to FDs' opinions. It is hard for patients to see the true reasons for their problems and to accept the diagnosis of depression, as was shown also by Ralition et al [12]. Major problems for FDs were related to treatment of depression. FDs believed that treatment problems originated first from the patients' difficulties as well as from their own inadequate resources. Treatment is often time and resource consuming, patients tend to stop taking the prescribed medicine, or they do not procure it altogether; this finding was supported also by other authors [22]. Second, FDs admitted their insufficient skills in psychotherapy. Patients need psychotherapy in addition to medication to obtain good treatment results and sometimes only psychotherapy would be enough. The efficacy of psychotherapy for treatment of depression has been shown in primary care [23]. Although FDs receive training in communication skills, their knowledge of psychological counselling and psychotherapy is mainly theoretical. Thus it is not realistic to expect that FDs conduct psychotherapy themselves, they need not replace other specialists, nor do they have time for professional psychotherapy. But problems lie in the lack of cooperation with psychiatrists and psychologists and in difficulties of availability of psychotherapists, as FDs also mentioned. Psychiatric and psychological care is concentrated into four major cities. There is lack of psychologists and psychotherapists and most psychological service is not covered by health insurance.

Although the identified issues are far from being exhaustive and there is some overlap, the study suggests a framework for understanding the incentives and the worries that FDs have when treating depressive patients. The obtained knowledge could be beneficial for specialized education in the future.

It is characteristic of our study that FDs thought that continuous training was still necessary although most of them had passed advanced training in depression. For comparison, according to a study of Soykan, only those physicians interested in psychiatry had passed training in depression [19]. It shows that our FDs recognise the need for dealing with the problem and are often engaged in it in their daily work.

## Conclusion

FDs are ready to manage patients with depressive complaints. They find sufficient motivation for dealing with the patients' psychological problems in the doctor-patient relationship context, but they wish to receive additional training and would like to schedule their consultations in a way they could have more time for depressive patients.

## Abbreviations

FD – family doctor

## Competing interests

The author(s) declare that they have no competing interests.

## Authors' contributions

PÖ designed and carried out the study, performed statistical analysis and completed the manuscript. AA and RK participated in data analysis and in writing of the manuscript. HIM participated in designing the study, in data analysis and in writing of the manuscript. All authors have read and approved the submitted version of the manuscript.

## Appendix 1

1. Personal data:

Age_______

Sex _______

Length of service as a physician ________

Length of service as a family doctor _______

2. Type of practice: Solo ____ Group _____

3. Location: Rural _____ Urban _____

4. What problems do you meet in your everyday work with depressive patients?

5. Is management of depression your daily work? Yes ____ No ____

6. Are you ready to deal with depressive patients? Yes ____ No ____

7. If yes, what is you motivation to deal with depressive patients?

8. Do you deal with depression? Yes ____ No ____

9. Do you have sufficient knowledge to deal with depression?

10. Do you need further training to deal with depression? Yes ____ No ____

## Pre-publication history

The pre-publication history for this paper can be accessed here:


